# Effects of Galvanic Vestibular Stimulation on Upper and Lower Extremities Motor Symptoms in Parkinson’s Disease

**DOI:** 10.3389/fnins.2018.00633

**Published:** 2018-09-11

**Authors:** Mahta Khoshnam, Daniela M. C. Häner, Eunice Kuatsjah, Xin Zhang, Carlo Menon

**Affiliations:** ^1^Menrva Research Group, Schools of Mechatronic Systems and Engineering Science, Simon Fraser University, Burnaby, BC, Canada; ^2^ETH Zürich, Zurich, witzerland

**Keywords:** Parkinson’s disease, galvanic vestibular stimulation, iTUG test, gait analysis, finger tapping task

## Abstract

As a neurodegenerative movement disorder, Parkinson’s disease (PD) is commonly characterized by motor symptoms such as resting tremor, rigidity, bradykinesia, and balance and postural impairments. While the main cause of PD is still not clear, it is shown that the basal ganglia loop, which has a role in adjusting a planned movement execution through fine motor control, is altered during this disease and contributes toward the manifested motor symptoms. Galvanic vestibular stimulation (GVS) is a non-invasive technique to influence the vestibular system and stimulate the motor system. This study explores how the motor symptoms of upper and lower extremities in PD are instantly affected by vestibular stimulation. In this regard, direct current GVS was applied to 11 individuals with PD on medication while they were performing two sets of experiments: (1) Instrumented Timed Up and Go (iTUG) test and (2) finger tapping task. The performance of participants was recorded with accelerometers and cameras for offline processing of data. Several outcome measures including coefficient of variation of the step duration, gait phase, phase coordination index, tapping score, and the number and duration of manual motor blocks (MMBs) were considered for objective quantifying of performance. Results showed that almost all of considered outcome measures were improved with the application of GVS and that the improvement in the coefficient of variation of the step duration, the tapping score, and the number of MMBs was statistically significant (*p*-value < 0.05). The results of this study suggest that GVS can be used to alleviate some of the common motor symptoms of PD. Further research is required to fully characterize the effects of GVS and determine its efficacy in the long term.

## Introduction

Parkinson’s disease (PD) is the second most common neurodegenerative disease (Hirtz et al., 2007) that is often accompanied by degradation of motor performance manifested by symptoms such as tremor, rigidity, bradykinesia, akinesia (especially of the face), postural instability, and freezing episodes (Lee et al., 2015). The affected individuals also experience cognitive difficulties, sleep disorder, and sensory deficits (Litvan et al., 2012). A number of observed symptoms are associated with a disturbed ‘internal’ clock leading to a different perception of rhythmic patterns (Ziv et al., 1999; Yahalom et al., 2004). Since the underlying mechanisms of many of the motor symptoms of PD are still not well understood, there is a lack of effective treatment options and the focus is on managing the symptoms, mainly with medication, to maintain or improve quality of life. Considering that the progression of PD involves the degeneration of dopaminergic neurons in the substantia nigra resulting in dopamine depletion in the striatum, the prescribed medication often includes a dopamine agonist such as Levodopa. However, it has been shown that medications have limited effects and their long-term use might cause undesirable side effects (Thanvi et al., 2007; Obeso et al., 2008; Nieuwboer and Giladi, 2013). While surgical interventions such as deep brain stimulation have also been used to manage some of the symptoms, their invasive aspect, the possibility of post-surgery complications, and neuropsychiatric side effects have limited their application (Benninger and Lomarev, 2010). Non-invasive interventions such as transcranial direct current stimulation have been investigated as alternative therapeutic methods (Nitsche et al., 2008).

Galvanic vestibular stimulation (GVS) is a non-invasive technique to stimulate vestibular nerves through electrodes that are placed over mastoid bones behind the ears. The current stimulus can be applied in different waveforms such as white noise, pink noise, or direct current. The vestibular nerve projects from underneath the mastoid bone to the cerebellar vermis through which the basal ganglia and the limbic system are activated, and the dopamine and noradrenaline levels are altered (Pan et al., 2008; Kataoka et al., 2015). Consequently, it has been shown that in neurodegenerative disorders such as PD, GVS can improve motor functions (Yamamoto et al., 2005; Pan et al., 2008; Lee et al., 2015; Samoudi et al., 2015). The effect of GVS on postural stability and balance has also been reported (Pal et al., 2009; Kataoka et al., 2015; Samoudi et al., 2015).

It is well-established that altered posture, reduced balance, and abnormal gait patterns are common functional disorders experienced by individuals with PD (Rogers, 1996; Yogev et al., 2005). Noisy subthreshold GVS has been shown to positively affect the gait of healthy subjects by decreasing gait asymmetry and improving the bilateral phase synchronization (Wuehr et al., 2016). The effects of supra-threshold GVS on walking trajectory of healthy subjects have also been documented (Bent et al., 2000). However, while GVS has been shown to reduce postural instability and sway (Pal et al., 2009; Kataoka et al., 2015) and improve balance (Samoudi et al., 2015) in PD, its effects on gait characteristics have not yet been studied in PD population.

To investigate the motor deficits in upper extremities of PD individuals, either unimanual or bimanual rhythmic tasks were administered: It has been shown that individuals with PD have more difficulty maintaining a prescribed rhythm in a finger tapping task (Freeman et al., 1993) and demonstrate more frequent manual motor blocks (MMBs) while tapping their finger (Ziv et al., 1999). Moreover, there is evidence that MMBs are correlated with freezing of gait, suggesting that the motor deficits of upper and lower limbs might share similar underlying mechanisms (Nieuwboer et al., 2009). The effects of GVS on performing manual rhythmic tasks have not yet been documented.

This study investigates the instant effects of supra-threshold GVS on gait characteristics and manual motor performance of individuals with PD. To the best of authors’ knowledge, such a study is unprecedented in the literature. 11 volunteers with PD participated in the study and completed a set of walking trials as well as a set of rhythmic finger tapping trials. Several outcome measures were defined to assess the performance with and without the application of GVS. Results show that the application of GVS improves motor performance in both upper and lower limbs.

## Materials and Methods

### Participants

Participants in this study were required to have a clinically confirmed diagnosis of PD, have a Mini Mental State Examination (MMSE) score of at least 26 points, and be able to walk independently (walking aids such as canes were allowed). Individuals who were not able to sit or stand unsupported, had other neurological disorder or heart conditions, had a vestibular disorder, or were enrolled in another research study involving drugs or devices were excluded. Based on these inclusion/exclusion criteria, eleven PD individuals who had moderate disease severity participated in this study (**Table 1**). Out of these participants, five individuals self-reported experiencing mild phases of freezing of gait. The study session for all subjects included conducting MMSE test and UPDRS (Unified Parkinson’s Disease Rating Scale) test 1 h after the subject had taken their medication.

**Table 1:** Summary of participants’ information.

Subject no.	Sex	Age (years)	Duration of the disease (years)	Freezer	MMSE score	UPDRS score	More affected side	Cutaneous sensory threshold (mA)
A0001	M	64	7	Yes	29	10	Left	0.75
A0002	F	61	19	Yes	26	20	Left	0.58
A0003	M	65	7	Yes	30	12	Left	0.28
A0006	F	67	5	No	28	7	Left	0.16
A0007	F	69	9	Yes	27	11	Left	0.06
A0008	M	72	17	No	28	20	Right	0.22
A0016	M	64	5	No	30	5	Right	0.30
A0017	F	81	22	Yes	30	13	Left	0.15
A0018	M	58	1/2	No	30	6	Right	0.26
A0019	M	64	1/2	No	29	20	Right	0.32
A0020	M	72	7	No	30	9	Left	0.74
Mean		67	9		28.8	12.1		


The protocol of this study was approved by the Office of Research Ethics at Simon Fraser University and all participants signed an informed consent form.

### Galvanic Vestibular Stimulation

For each participant, 2-inch round cloth neurostimulation electrodes (ValuTrode^®^, Axelgaard Manufacturing Co., Ltd., Fallbrook, CA, United States) were placed behind ears over the mastoid processes on both sides (cathodes) and on the proximal, medial section of both forearms (anodes). In order to improve skin-electrode contact and avoid skin irritation, skin surface at the electrode sites was cleaned with 70% alcohol prep pad (Covidien Curity, United States). The Linear Isolated Stimulator (A395R, World Precision Instruments Inc., Sarasota, FL, United States) was used for applying the electrical current to the vestibular system through the electrodes. The cutaneous sensory threshold was determined by slowly increasing the current intensity (Lee et al., 2015): starting from a base current level of zero, the current was applied with a stepwise 10 μA increase with an adjustment period of 20 s every two steps until the participant reported a tingling sensation at the electrode sites. The current level was then lowered to zero. After a rest period of 30 s to ensure that the effects of GVS application do not carry over to the confirmation test, this test was repeated for a second time to confirm the obtained cutaneous sensory threshold. The stimulus for “GVS on” tests was then set to a direct current equal to twice of the determined sensation threshold (**Table 1**). A ramp up and ramp down time of 3 s was administered to avoid discomfort when the stimulus was switched on and off.

### Study Protocol

The study was administered in one 2-h session. The participants were asked to be present in the research lab about 30 min after they had taken their medication. The study was then explained, their informed consent was collected, and the MMSE assessment was completed. The UPDRS assessment was started 1 h after the medication. The cutaneous sensory threshold was then determined, and a rest period of 5 min was allowed before beginning the tests. In order to investigate how the motor performance in upper and lower extremities is affected by GVS, the study consisted of two parts: (1) instrumented Timed Up and Go test and (2) finger tapping task.

#### Instrumented Timed Up and Go Test

During this walking test, the participants were asked to perform a 5-m Timed Up and Go (TUG) test at their preferred walking speed while wearing a pair of accelerometers (Physilog^®^ IV, GaitUp, Switzerland) fixed on the top of their shoes. A total of six iTUG trials were administered of which the first three were without stimuli (“GVS off” trials) and the last three were with vestibular stimulation (“GVS on” trials). Such an order for trials was chosen to minimize any residual effect that vestibular stimulation might have in the subsequent trials. To remove any bias that subjects might have had toward the electric sensation, the study was designed as a single-blind experiment in which the subjects were not aware of the order of “GVS on” and “GVS off” tests. The electrodes were placed on participants’ skin and were connected to the stimulator during the entire data collection session. Sham stimulation was administered during the “GVS off” trials. The participants were told that the stimulus was always on with different degrees of intensity and, therefore, they might or might not feel the current during the trials. The iTUG trials were timed with a stop watch and also recorded with a camera (EOS Rebel T1i, Canon, Japan).

#### Finger Tapping Task

**FIGURE 1:**
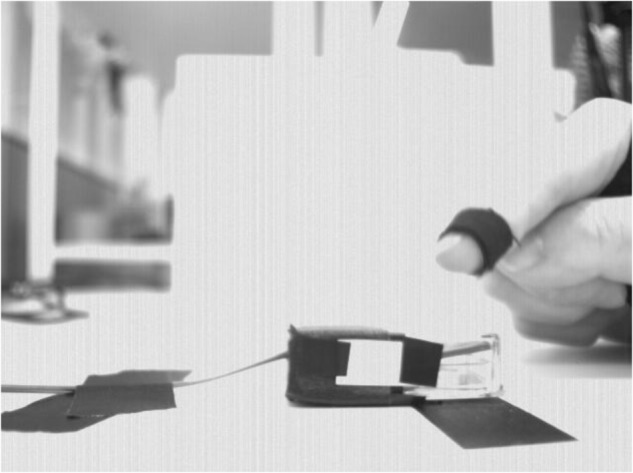
Sample of collected images during the finger tapping task. The black cotton kinesiology tape wrapped around the index finger was used to automatically track the position of the fingertip.

During this test, the participants were instructed to tap with the tip of their index finger on a block affixed on the desk surface. To ensure that the tapping was performed out of the wrist, the thumb pinched the index finger to support its position (**Figure 1**). The subjects were asked to tap at their preferred speed while trying to maintain the same rhythm for 20 s. A total of six tapping trials was recorded from each participant of which the first three trials were without applying the stimulus (“GVS off” trials) and the last three were administered with vestibular stimulation (“GVS on” trials). Each trial consisted of two sub-trials of a tapping task with the right hand and a task with the left hand performed successively with a 3-min rest period in between trials. Similar to the iTUG test, in order to blind subjects with respect to the testing condition, the participants were told that the current stimulus is always applied but with different degrees of intensity. A high-speed camera (Fastec Imaging Corporation, San Diego, CA, United States) was used to record images of trials with a resolution of 640 × 480 at 125 frames per second.

### Outcome Measures

All data collected during a study session were analyzed offline after the session had been concluded. Several outcome measures were considered for the iTUG tests and finger tapping tasks as explained in this section. **Table 2** provides a summary of the defined outcome measures and their abbreviations.

**Table 2:** Summary of the defined outcome measures.

Test	Outcome measure	Abbreviation	Description
iTUG	Coefficient of variation of the step duration	Tstep CV	The level of consistency in the step duration.
iTUG	Phase	Phase	The similarity between swing times of right and left legs.
iTUG	Phase coordination index	PCI	The accuracy and consistency of generating gait phases.
iTUG	Tremor index	TI	The amount of foot tremor during the walking tests.
Finger tapping	Coefficient of variation of inter-tap time	Intertap CV	The periodicity of the tapping.
Finger tapping	Number of manual motor blocks	Number of MMBs	The ability to maintain the tapping rhythm.
Finger tapping	Duration of manual motor blocks	Duration of MMBs	The ability to maintain the tapping rhythm.
Finger tapping	Tapping score	TS	Speed and inter-tap consistency of the tapping.
Finger tapping	Tapping tremor index	TTI	The amount of finger tremor during the walking tests.


#### Instrumented Timed Up and Go Test

The data for the iTUG test were saved in Physilog accelerometers and were transferred to a computer after the session to be further processed in MATLAB (The MathWorks, Inc., Natick, MA, United States). Since the iTUG test included sit-to-walk and walk-to-sit phases as well, the acceleration signal was trimmed to only include the period of regular gait. Gait cycles were then determined from the acceleration signals and were confirmed with video recordings of the trial following the method described in Jimenez et al. (2009). Briefly, the local acceleration variance was calculated based on the magnitude of the acceleration signal. A threshold was applied to distinguish between lower values (flat foot phase) and higher values (swing phase). The beginning of flat foot phases was then defined as the time points at which a gait cycle starts. The following outcome measures were calculated based on the acceleration signal and the determined gait cycles.

##### Coefficient of variation of the step duration (Tstep CV)

This measure defined in (Plotnik et al., 2007) quantifies the level of consistency in the step duration as an index of bilateral coordination of gait and is expressed as a percentage ratio by dividing the standard deviation of step duration by the mean step duration. Therefore, a lower Tstep CV indicates a higher uniformity in the timing of steps.

##### Phase

Phase is also a measure of bilateral gait coordination introduced in (Plotnik et al., 2007). To calculate this measure, the average of swing times for both legs are first calculated. If the duration of each step corresponds to 360°, the phase is defined by calculating the ratio between the difference in average swing times and the duration of step. With such a definition, if the gait is symmetrical, both right and left legs will have similar swing times and, therefore, the phase will be equal to 180°. A phase value that is far from 180° shows asymmetrical and abnormal gait patterns.

##### Phase coordination index (PCI)

Defined in Plotnik et al. (2007), phase coordination index (PCI) is another measure of bilateral gait coordination to quantify the accuracy and consistency of generating gait phases. This measure indicates the level of variations in phase during consecutive steps and is expressed as a percentage value. A lower PCI denotes a higher consistency in gait phase generation.

For a more detailed explanation of Tstep CV, phase, and PCI and their mathematical expressions, please see (Plotnik et al., 2007; Palmerini et al., 2013).

##### Tremor index (TI)

Frequency characteristics of acceleration signals have been previously used to objectively detect episodes of freezing of gait (Moore et al., 2008, 2013). As reported in those studies, in normal gait patterns, the vertical acceleration signal includes components with lower frequency and, therefore, the signal has higher power in the 0–3 Hz frequency band, named the “locomotor band.” During episodes of freezing of gait, the trembling of the legs results in higher signal power in the 3–8 Hz frequency band, namely the “freeze band.” The ratio between the squared area under the power spectrum in the freeze band and that in the locomotor band was then used in the detection of freezing episodes. In the current study, a similar idea has been implemented by defining the Tremor Index (TI) of an iTUG trial by dividing the square of the area under the power spectrum in the 3–8 Hz band by that in the 3–8 Hz band. A lower TI denotes less tremor during the walking test.

#### Finger Tapping Task

To facilitate extracting the position of the fingertip during the tapping trials, a black cotton kinesiology tape (Beschoi, China) was wrapped around the tip of the index finger and a white background was arranged (**Figure 1**). A custom MATLAB script was developed to automatically determine the position of the fingertip in each acquired frame.

##### Coefficient of variation of inter-tap time (Intertap CV)

This measure is an indication of the periodicity of the tapping. A lower Intertap CV shows less variations in the time interval between the taps and, therefore, indicates a better performance.

##### Number and duration of manual motor blocks (MMBs)

These measures show the ability to maintain the rhythm throughout the internally cued manual tapping task. Introduced in Ziv et al. (1999), a MMB is defined as an interruption in the rhythm that is longer than the sum of the mean inter-tap interval and two times of the inter-tap standard deviation. Duration of MMBs is expressed as a percentage value with respect to the total time of the tapping task.

##### Tapping score (TS)

This measure introduced in Jobbágy et al. (2005) takes into account both speed and inter-tap consistency of the tapping task to calculate a tapping score (TS) for each tapping trial. A higher TS value is an indication of better performance.

##### Tapping tremor index (TTI)

This measure is defined herein based on the frequency characteristics of the fingertip acceleration during the tapping task. To calculate this measure, the tapping frequency (*f_tap_*) is first determined. The tapping tremor index (TTI) is defined as the ratio between the squared area under the power spectrum of acceleration in the frequency band with frequencies greater than 1.2*f_tap_* and that in the 0.8*f_tap_*–1.2*f_tap_* frequency band. A lower TTI value shows that the majority of power occurs in the tapping frequency band and, therefore, indicates a better performance.

## Results

The outcome measures defined in Section “Outcome Measures” were calculated for the data collected during the walking and finger tapping tasks. Considering that 11 subjects participated in the study and each subject completed three “GVS off” and three “GVS on” trials, each of the outcome measures described in Section “Outcome Measures” contained 33 samples for each of these two cases. A Chi-square goodness-of-fit test was used to determine if an outcome measure was normally distributed. If the normal distribution of that outcome measure was confirmed, a *t*-test was used to determine if GVS had a significant effect on the considered measure. Otherwise, the signtest was used to determine the significance of the GVS effect. All statistical analysis was done using Statistics and Machine Learning Toolbox included in MATLAB. A significance level of 0.05 was considered and the null hypotheses were that (1) applying GVS does not affect the gait parameters, and (2) applying GVS has no effects on the periodicity of finger taps. This section summarizes the study results.

### Instrumented Timed Up and Go Test

The Tstep CV was found to be 14.75 ± 5.51 in “GVS off” trials and 12.34 ± 4.17 in “GVS on” trials which indicates 16% reduction in variations of the step duration with GVS application.

The phase value was 191 ± 56.7 in “GVS off” trials and 193.71 ± 47.2 in “GVS on” trials. Since the value of phase for normal gait is close to 180°, this result shows slight deterioration of gait (1.4%) with GVS application, although this did not reach significance.

The PCI for “GVS off” trials was calculated to be 27.57 ± 36.67 and for “GVS on” trials was 24.99 ± 36.64. This result shows that the gait consistency was improved by 9.3% with GVS application.

The TI was 0.16 ± 0.12 for “GVS off” trials and 0.14 ± 0.09 for “GVS on” trials which shows 12.75% reduction in high-frequency tremors of the feet with GVS application. This measure was also evaluated for each foot separately: The TI for the more affected side was 0.19 ± 0.14 in “GVS off” trials and 0.17 ± 0.11 in “GVS on” trials, i.e., 11.6% reduction in tremor while walking with GVS application. The TI for the less affected side was found to be 0.19 ± 0.14 in “GVS off” trials and 0.17 ± 0.11 in “GVS on” trials which is equivalent to 14.4% improvement in performance.

**FIGURE 2:**
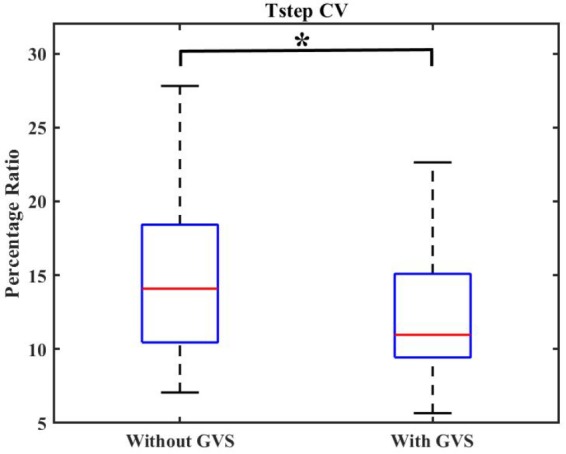
Boxplot demonstrating the effect of GVS on Tstep CV. The symbol ^∗^ indicates statistical significance with the two groups.

The significance of the obtained results was evaluated by statistical analysis. The obtained *p*-values are reported in **Table 3**. **Figure 2** shows a sample of boxplots illustrating the results.

**Table 3:** *p*-values calculated for the outcome measures defined for the iTUG tests (Tstep CV, coefficient of variation of the step duration; PCI, phase coordination index; TI, tremor index).

Outcome measure	*p*-value
Tstep CV	0.046
Phase	0.486
PCI	0.080
TI	Both sides: 0.064
	More affected side: 0.396
	Less affected side: 0.604


### Finger Tapping Task

The Intertap CV was calculated to be 65.49 ± 46.15 in “GVS off” trials and 54.48 ± 27.49 in “GVS on” trials for the more affected side, i.e., 16.8% improvement in the consistency of the tapping rhythm with GVS application. The Intertap CV for the less affected side was found to be 45.38 ± 25.42 in “GVS off” trials and 46.82 ± 21.64 in “GVS on” trials which the rhythm is slightly (3.1%) deteriorated with applying GVS. Considering the performance of both hands, the Intertap CV in “GVS off” trials was 55.43 ± 38.33 and 50.65 ± 24.87 in “GVS on” trials which shows 8.6% improvement in rhythmicity.

The number of MMBs was 1.42 ± 1.22 for “GVS off” trials and 1.45 ± 1.2 for “GVS on” trials for the more affected side which shows 2% reduction in the rhythm interruption with GVS application. The number of MMBs for the less affected side was found to be 1.79 ± 1.65 in “GVS off” trials and 1.33 ± 1.49 in “GVS on” trials which indicates 25.7% improvement in tapping rhythm with GVS application. If the performance of both hands is considered, the number of MMBs was 1.62 ± 1.44 for “GVS off” trials and 1.38 ± 1.36 for “GVS on” trials which indicates 15% reduction in the number of MMBs with GVS application.

The duration of MMBs was 3.16 ± 2.46 for “GVS off” trials and 3.05 ± 2.55 for “GVS on” trials for the more affected side which is equivalent to 3.5% reduction in the length of rhythm interruptions with GVS application. The duration of MMBs for the less affected side was found to be 3.13 ± 2.42 in “GVS off” trials and 2.3 ± 2.25 in “GVS on” trials which shows 26.5% reduction in the length of rhythm interruptions. For both hands, the duration of MMBs was 3.14 ± 2.42 for “GVS off” trials and 2.67 ± 2.41 for “GVS on” trials, equivalent to 15% reduction in the duration of MMBs with GVS application.

The TS was 24.78 ± 18.53 for “GVS off” trials and 31.18 ± 23.68 for “GVS on” trials for the more affected side which shows 28.25% improvement in speed and rhythmicity of taps with GVS application. For the less affected side, the TS was found to be 26.35 ± 17.14 in “GVS off” trials and 32.47 ± 20.67 in “GVS on” trials which indicates 23.2% improvement with GVS application. Considering the performance of both hands, the TS was 25.57 ± 17.73 for “GVS off” trials and 31.82 ± 22.06 for “GVS on” trials, equivalent to 24.5% improvement in performance with applying GVS.

The TTI was 153.46 ± 293.24 for “GVS off” trials and 141.61 ± 240.88 for “GVS on” trials for the more affected side which shows 7.7% reduction in high-frequency tremors of finger with GVS application. The TTI for the less affected side was found to be 190.58 ± 489.11 in “GVS off” trials and 109.75 ± 210.5 in “GVS on” trials which indicates 42.4% improvement in tremor reduction. Regarding the performance of both hands, the TTI was 172.02 ± 400.57 for “GVS off” trials and 125.68 ± 225.03 for “GVS on” trials indicating 26.9% reduction in high frequency tremors with GVS application.

**FIGURE 3:**
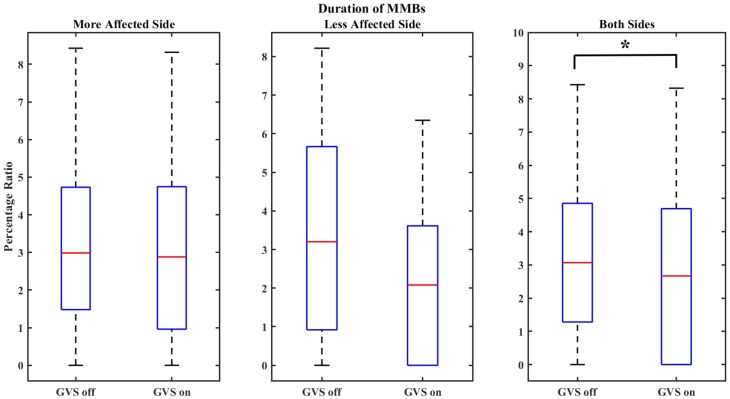
Boxplot demonstrating the effect of GVS on the duration of MMBs. The symbol ^∗^ indicates statistical significance with the two groups.

**FIGURE 4:**
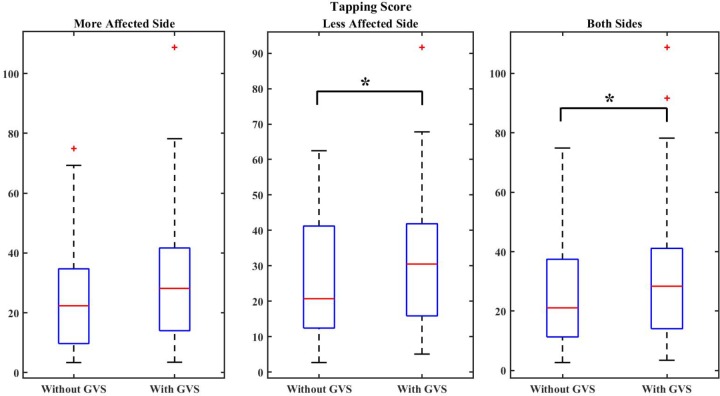
Boxplot demonstrating the effect of GVS on the TS. The symbol ^∗^ indicates statistical significance with the two groups.

*p*-values were calculated to evaluate the significance of the obtained results as reported in **Table 4**. **Figures 3**, **4** show samples of boxplots illustrating the results.

**Table 4:** *p*-values calculated for the outcome measures defined for the finger tapping task (Intertap CV, coefficient of variation of inter-tap time; Number of MMBs, number of manual motor blocks; Duration of MMBs, duration of manual motor blocks; TS, tapping score; TTI, Tapping tremor index).

Outcome measure	*p*-value		
	
	More affected Side	Less affected side	Both sides
Intertap CV	0.296	0.718	0.712
Number of MMBs	0.541	0.096	0.072
Duration of MMBs	0.867	0.089	0.013
TS	0.073	0.044	0.007
TTI	0.816	0.324	0.267


## Discussion

The study presented herein is a step toward understanding the effects of GVS on motor performance of individuals with PD. In this paper, two separate protocols were designed to assess the effects of GVS on the performance of both upper and lower extremities. Several outcome measures were defined to capture both temporal and frequency characteristics of movements. These outcome measures were chosen from literature to objectively quantify the walking and tapping performance. Common clinical assessments, such as the UPDRS test score, were not considered as outcome measures due to their relying on rater’s observation.

The 5-m iTUG test was administered to characterize the effects of GVS on the quality of gait generation and bilateral coordination. Although the TUG test commonly consists of a 3-m course, an extended course of 5 m was considered in this study to have longer periods of regular gait after discarding the data corresponding to sit-to-walk and walk-to-sit phases (Palmerini et al., 2013). TUG tests are commonly used to assess a person’s mobility. The iTUG test time was recorded for each trial and, as reported in **Table 5**, was generally faster in “GVS on” trials. However, since the participants were asked to complete the trials at their preferred speed, the time taken to complete an iTUG test was not defined as an outcome measure. Evaluating several temporal and frequency-based outcome measures showed that the timing of steps was significantly improved with applying GVS. Although a more extensive study is required for significant results, a positive effect of GVS on bilateral gait coordination and tremor reduction was shown through decreases in the PCI and the TI values.

**Table 5:** Time (s) taken to complete iTUG trials.

Subject no.	GVS off			GVS on			Mean	
			
	Trial 1	Trial 2	Trial 3	Trial 1	Trial 2	Trial 3	GVS off	GVS on
A0001	19.65	17.07	15.22	15.38	15.22	15.25	17.31	15.28
A0002	15.22	15.13	14.38	15.63	14.94	15.56	14.91	15.38
A0003	16.22	15.68	15.12	15.57	13.37	14.00	15.67	14.31
A0006	11.56	11.35	10.94	11.10	10.69	10.26	11.28	10.68
A0007	20.75	17.56	17.72	17.50	16.22	17.25	18.68	16.99
A0008	20.47	18.90	23.12	20.81	20.91	20.22	20.83	20.65
A0016	20.59	17.88	18.03	18.35	16.91	16.06	18.83	17.11
A0017	16.64	14.90	14.15	15.15	14.22	13.64	15.23	14.34
A0018	17.56	14.98	14.02	14.03	13.38	13.61	15.52	13.67
A0019	22.09	19.08	19.56	19.76	19.50	14.41	20.24	17.89
A0020	15.97	16.09	14.63	15.50	13.22	12.56	15.56	13.76
Mean	17.88	16.24	16.08	16.25	15.33	14.80	16.73	15.46


Since PD interferes with generating rhythmic movements by disrupting the ‘internal clock’ (Ziv et al., 1999; Yahalom et al., 2004), an internally cued finger tapping task was designed to assess the effects of GVS on the periodicity of movement generation. The duration of each finger tapping trial was chosen to be 20 s from which the mid 15 s were chosen for analysis. As reported in Ziv et al. (1999), 15 s are enough to induce fatigue such that MMBs are observed. Applying GVS significantly reduced the duration of MMBs and significantly improved the TS which is an index combining both speed and rhythmicity of taps. We also showed a positive trend toward reducing the number of MMBs. The positive effect of GVS on MMBs was observed in all subjects regardless of the stage or duration of the disease. Although not significant, high-frequency tremors of the fingertip were reduced with application of GVS.

Considering that individuals with PD experience some degrees of vestibular dysfunction (Reichert et al., 1982), application of GVS acts as a medium that increases gait stability and coordination by affecting vestibular afferents (Wuehr et al., 2016). Moreover, since rhythm and fine motor control required to follow a rhythmic movement such as tapping a finger is modulated in the basal ganglia loop, the improved performance observed in this study might be attributed to GVS influencing the striatum through the afferents of the vestibular nerve. It has been previously shown that individuals with PD are able to maintain the finger tapping rhythm better if they initialize their tapping by synchronizing their movements with external rhythmic auditory cues (Freeman et al., 1993). The results of our study show that GVS can assist with self-paced rhythmic movement generation without requiring external cues, further promoting the idea that GVS might affect the ‘internal clock.’

When applicable, the defined outcome measures were calculated for the less affected side and the more affected side separately. The results showed that in the majority of cases, GVS improves the performance in both sides. The only exception to this result was the periodicity of the tapping (Intertap CV) which was slightly worse with applying the stimulus. Further study is required to determine if GVS significantly affects one side more than the other. It is also worth noting that the duration of the disease might influence the results of GVS application. To investigate this point, statistical analysis should consider the participants in two subgroups according to their disease duration: individuals with longer disease duration and individuals with shorter disease duration. Unfortunately, in this study, the number of subjects was not sufficient to obtain significant results for GVS application in each subgroup, but this point will be considered in future studies.

This study was designed to assess the instant effects of supra-threshold direct current GVS on gait features and generation of rhythmic movements. Although no long-lasting effects were reported using low-intensity GVS (Ferrè et al., 2013), to the best of authors’ knowledge, there have been no studies investigating how long the effects of GVS last in PD individuals. The “GVS on” trials were administered after the “GVS off” trials to avoid carrying on any residual effects of GVS application to “GVS off” trials. Nevertheless, considering the single-blind design of the study and according to the feedback from the participants, the subjects were not aware of the order of tests and were under the impression that the current stimulus was always being applied. Interestingly, when being asked after the data collection session, participants could not comment about the intensity of current or tell which of the trials were “GVS on” ones. This might be due to that when measuring the threshold, the participants were focused on perceiving the tingling sensation at the electrode sites, but during the tests they were concentrating on completing the administered task. The participants were asked to complete a questionnaire at the end of the study session. **Table 6** summarizes the results of this questionnaire. Overall, the participants did not report any side effects such as discomfort or dizziness. Moreover, no sway or deviation from the intended walking path was observed. No cases of discomfort or dizziness were reported after the sessions were concluded and all participants were able to finish complete sets of the iTUG and the finger tapping task.

**Table 6:** Participants’ answers to the questionnaire completed at the end of the study session: Q1: I felt dizzy or pain during the trials; Q2: I have sensitivity of the skin where the GVS electrodes were placed; Q3: I am interested in participating in later phases of this study (1: Strongly Disagree; 2: Disagree; 3: Neither Disagree or Agree; 4: Agree; 5: Strongly Agree).

Subject no.	Q1	Q2	Q3
A0001	1	2	5
A0002	1	1	5
A0003	1	1	5
A0006	1	1	5
A0007	1	1	4
A0008	2	2	4
A0016	2	2	4
A0017	1	1	5
A0018	1	1	5
A0019	1	2	5
A0020	1	1	5
Mean	1.18	1.36	4.73


Although five out of 11 participants reported experiencing symptoms of freezing of gait, no freezing episodes were encountered during the iTUG trials. This might be due to the fact that eliciting freezing of gait is more difficult in the laboratory setting (Snijders et al., 2008). It has been shown that MMBs are correlated with the occurrence of freezing of gait (Ziv et al., 1999; Nieuwboer et al., 2009). Since both finger tapping and gait generation might be considered as rhythmic movements, such a correlation along with the results obtained in this study might be another piece of evidence that applying GVS improves gait performance. The results of this study are also in accordance with those reported in Wuehr et al. (2016) which conclude that noisy GVS improves walking stability in healthy population. In the study herein, similar results were obtained since less variations of gait and better PCI were observed with applying DC GVS to individuals with PD.

As a preliminary investigation, this study had certain limitations that can be addressed in future research. Testing on a larger group of individuals with PD is required to establish significant results for all of the considered outcome measures. Moreover, other outcome measures that characterize the quality of gait and rhythmic movement generation can be defined. The current stimulus in this study was chosen to be a direct current equal to two times the cutaneous sensory threshold delivered to mastoid bones and forearms to investigate the effects of such a stimulus. Other current levels and placement of electrodes might be explored as well. Moreover, since the anodes were placed on the forearms, it is expected that applying GVS creates a forward swing (Magnusson et al., 1990; Day et al., 2011). However, no forward swing was observed during the testing or afterward when re-examining the recorded sessions. A sophisticated motion tracking system can be used to record the movements of all limbs and determine if such a sway occurs particularly during the walking tests and if it helps with step initiation and improving the rhythm of step generation. The study presented herein was administered in one session. Further multi-session studies are required to establish how individuals with PD adapt to current stimulation in order to determine the efficacy of GVS in long term.

## Conclusion

This study investigated the instant effects of supra-threshold GVS using direct current on gait parameters and internally cued rhythm generation of individuals with PD. In this regard, 11 participants were instructed to complete a series of iTUG trials and finger tapping trials without and with GVS. The acceleration signal of the feet during the iTUG tests and the fingertip trajectory during the finger tapping task were recorded and processed to quantify the effects of GVS. Results showed that application of GVS significantly improves the timing of steps and has an almost significant effect on the consistency of gait phase generation. Moreover, applying GVS significantly improved the speed and rhythmicity of finger tapping and significantly decreased the duration of MMBs and almost significantly reduced the number of interruptions in the tapping rhythm. The obtained results suggest the positive influence of GVS on gait characteristics as well as internally cued rhythmic movements.

## Ethics Statement

Thisstudy was carried out in accordance with the recommendations of Office of Research Ethics at Simon Fraser University. The protocol was approved by the Office of Research Ethics at Simon Fraser University. All subjects gave written informed consent in accordance with the Declaration of Helsinki.

## Author Contributions

MK, DH, and CM conceived and designed the experiments. EK implemented the experimental setup. DH and EK collected the data. DH, MK, and XZ analyzed the data. MK and DH wrote the manuscript.

## Conflict of Interest Statement

The authors declare that the research was conducted in the absence of any commercial or financial relationships that could be construed as a potential conflict of interest.
